# Advancing epidemic intelligence: evaluating Senegal’s mpox surveillance system and readiness for AI-driven predictive modelling

**DOI:** 10.3389/fpubh.2026.1742888

**Published:** 2026-02-03

**Authors:** Sylvain L. B. Faye, Fatoumata B. Diongue, Abdourakhmane Ndao, Boly Diop, Georgette H. C. Sow, Ndiaye Dia, Fallou Diakhate, Tidiane Gadiaga, Pape Samba Dieye, Oumou Kalsom D. Gueye, Yoro Sall, Ibrahima Seck, Youssou Bamar Gueye, Aminata Massaly, Moussa Seydi, Ibrahima Sy

**Affiliations:** 1 Laboratory of Sociology, Anthropology, and Psychology (LASAP-ETHOS) FLSH, Cheikh Anta DIOP University – DAKAR, Dakar, Senegal; 2 Institute of Health and Development (ISED), FMPOS, Cheikh Anta DIOP University, Dakar, Senegal; 3 Epidemiological Surveillance and Vaccination Response Division, Ministry of Health and Public Hygiene (MSHP), Dakar, Senegal; 4 S&F Pro Consulting, Selangor, Malaysia; 5 Senegalese Institute of Algorithms (IAS), Dakar, Senegal; 6 Health and Social Information System Division (DSISS), Ministry of Health and Public Hygiene (MSHP), Dakar, Senegal; 7 Center for Health Emergency Operations (COUS), Ministry of Health and Public Hygiene (MSHP), Dakar, Senegal; 8 Infectious and Tropical Diseases Department (SMIT), Fann Hospital, Dakar, Senegal; 9 Ministry of Health and Public Hygiene (MSHP), Dakar, Senegal

**Keywords:** artificial intelligence, epidemiological surveillance, mpox, predictive modeling, West Africa

## Abstract

**Introduction:**

Mpox has re-emerged as a public health issue in West Africa, underscoring the need for robust surveillance systems that can detect outbreaks and facilitate effective responses. This study evaluates Senegal’s mpox surveillance system, focusing on performance, data quality, governance, and potential for Artificial Intelligence-powered, predictive epidemic intelligence. It reviews trends and system operations while exploring AI and modeling to improve early warnings.

**Methodology:**

A descriptive, exploratory approach combined quantitative and qualitative data from various sources. A retrospective review of mpox cases from January 2024 to October 2025 utilized DHIS2 Tracker to analyze geographical, temporal, and demographic patterns, as well as reporting delays and biases. Data-quality checks and stakeholder interviews provided insights into system performance, intersectoral coordination, and preparedness for advanced analytics.

**Results:**

By late October 2025, Senegal had reported seven mpox cases, all in Dakar, primarily affecting young, mobile populations, with a higher incidence among children and working-age adults. Transmission followed population movement along the Dakar–Thiès–Diourbel corridor, showing how urban density and mobility influence spread. The surveillance system improved reporting, geolocation, and follow-up, supported by One Health coordination and digital health infrastructure. Challenges include underreporting in rural areas, uneven coverage, limited real-time analytics, and gaps in data interoperability and responsible AI regulation. The AI4MPOX-SN initiative offers an opportunity to enhance epidemic intelligence by integrating human-animal-environment data, using AI for anomaly detection and predictive modeling to inform interventions.

**Conclusion:**

To develop predictive epidemic intelligence in Senegal, it’s vital to involve local stakeholders, promote transparency, build workforce capacity, and establish safeguards for the ethical use of data. Combining technology, participatory governance, and institutional strengthening will enable Senegal to transition from reactive detection to proactive surveillance, positioning it as a regional leader in health security in West Africa.

## Introduction

Mpox, formerly monkeypox, is a zoonotic virus transmitted through close contact (1). Since 2022, it has been a public health emergency with outbreaks outside endemic areas (2). Between 2022 and 2024, the World Health Organization (WHO) reported over 124,700 cases and 272 deaths in 128 countries (3). In 2024, 15 African countries reported 15,992 cases and 60 deaths, with the Democratic Republic of Congo (DRC), Burundi, and Uganda accounting for 96% of cases and 97% of fatalities (4). Kenya, Rwanda, and Uganda reported cases for the first time, indicating a rapid spread and regional vulnerability to silent transmission and virus diversification (5). In West Africa, Mpox remains a significant public health challenge, with ongoing transmission supported by recent outbreaks and movement patterns. In Guinea, a resurgence began in mid-2025, with hundreds of confirmed cases primarily in Conakry and surrounding areas (4). Sierra Leone reported several thousand confirmed cases from 2024 to 2025, straining its health systems (4). Côte d’Ivoire has seen multiple isolated cases across new districts, indicating the spread of local transmission networks (4). Although Senegal reports fewer cases, it identified imported infections in August 2025 (6). Variations in notification timeliness and genomic data suggest possible localized transmission and geographic spread, highlighting regional vulnerability where mobility and cross-border travel may facilitate expansion if surveillance is not strengthened (7). These trends highlight persistent challenges related to underreporting, delayed case notification, and limited intersectoral data integration, underscoring the need for strengthened regional surveillance systems and coordinated cross-border response strategies (8).

Since the global Mpox outbreak in 2022, Senegal has taken deliberate steps to strengthen its epidemiological surveillance and response architecture. The country instituted event-based and entry-point monitoring systems in line with WHO Africa guidance, intensified case definition and contact tracing training, and launched targeted awareness campaigns in high-mobility urban settings (e.g., the 2024 Touba pilgrimage) (9). A retrospective, cross-sectional, descriptive, and analytical study (10) identified 100 suspected cases (6 per million), all of which were tested; however, no confirmed cases were found. Additionally, 32% presented with alternative diagnoses, primarily chickenpox and shingles, and 12.5% had co-infections. In August 2025, Senegal confirmed its first imported case, followed by an indigenous case of clade IIb in Dakar, indicating community circulation (11). After declaring a mpox emergency, Senegal improved its system by adopting the District Health Information Software 2 (DHIS2) Tracker for notification, case tracking, geolocation, and contact management.

An effective mpox response relies on thorough surveillance for early detection and rapid action. This includes active monitoring, reducing notification delays, improving response efficiency, adopting “One Health” approach, and coordinating efforts on cross-border movements and regional trade (12). In resource-limited countries like West Africa, surveillance faces challenges due to structural issues, including limited diagnostic capacity, reporting delays, difficulties in tracing, and underutilized data, which hinder decision-making, lead to underreporting, and slow the detection of outbreaks (13, 14). A recent study (11) highlights Senegal’s progress in surveillance, including the implementation of electronic notifications, sentinel sites, and laboratory strengthening. DHIS2 supports surveillance for aggregate and individual-level data and a One Health approach linking human, animal and environmental health sectors (15). Yet, data integration across human, animal, and ecological health remains limited, highlighting vulnerability even in the absence of major outbreaks. This underscores the need to enhance active surveillance, field epidemiology, and digital tools such as AI, modeling, and surveillance systems to improve response and data quality. Senegal’s public health system faces a pivotal question: can current mpox monitoring evolve into an AI-driven epidemic intelligence system?

Senegal is a promising setting for AI-driven surveillance because of strong national coordination and advancing digital health infrastructure. The Digital Health Strategic Plan 2018–2023 promotes interoperable systems, electronic patient records, and real-time data use (16) while the *New Deal Technologique Horizon 2034* sets a long-term vision for digital transformation across sectors, including public health (17). In March 2023, the World Bank approved a US$150 million grant to accelerate Senegal’s digital transformation, strengthening its legal frameworks, digital literacy, and access to services (18). The funding supports digital health innovations, including telemedicine, electronic medical records, and immunization management apps, as well as upgrades to broadband infrastructure. This is part of the Digital Economy Acceleration Project (PAEN), aligned with Axis I of the Emerging Senegal Plan (PSE), which aims to achieve emerging-economy status by 2035. The healthcare sector’s Health System Digitization Program encompasses six key initiatives: Shared Patient Records, telemedicine, hospital information systems, geographic health systems, drug digitization, and community health processes and governance digitization (19). These capabilities form a foundation for AI and predictive modeling, shifting from traditional reporting to proactive epidemic intelligence. Senegal transitions from isolated reporting to integrated, data-driven insights, enabling the testing of responsible AI tools to forecast outbreaks and inform swift decisions.

AI and predictive modeling are crucial for epidemic surveillance in Africa, enabling early detection, monitoring, and resource allocation (20–22). They analyze health data, mobility (23), and environmental factors to support targeted responses such as predicting hotspots (24). During the COVID-19 pandemic, AI improved models, accelerated vaccine development, and enabled real-time data-driven decision-making (25, 26). AI enhances situational awareness by recognizing patterns and adapting to dynamic environments (27), enabling proactive responses. Implementing AI can enhance preparedness, refine strategies, and improve health security, particularly in areas with limited infrastructure (28, 29). Building on these lessons, since 2025 the International Development Research Centre (IDRC) and the University of Toronto have implemented AI4Mpox (30), a multi-country initiative in seven mpox-affected African countries (Burundi, Cameroon, Democratic Republic of Congo, Ethiopia, Ghana, Nigeria, and Senegal). The project leverages AI, Big Data, and mathematical modeling to improve surveillance, preparedness, and response through locally contextualized, data-driven tools. Key outputs include real-time AI dashboards for outbreak monitoring; deep neural network–based optimization of interventions and vaccine distribution using community-level data; and enhanced surveillance and hotspot detection, building on COVID-19 analytical frameworks. Additionally, natural language processing techniques, such as topic modeling and sentiment analysis, are used to strengthen risk communication, counter misinformation, and reduce mpox-related stigma, enabling evidence-based decision-making and efficient health service delivery.

Looking ahead, integrating One Health data could further transform Senegal’s mpox reporting into a comprehensive, real-time system for detecting zoonotic spillovers. However, realizing this potential requires addressing data interoperability across human, animal, and environmental health domains, ensuring data quality, building technical expertise, and implementing ethical and confidentiality safeguards (31). Social acceptance and community engagement are also critical for responsible adoption (32). Evaluating Senegal’s current system against these criteria highlights both strengths and gaps, informing a phased roadmap toward AI-enhanced predictive epidemic intelligence that could serve as a model for other African contexts.

To build on this perspective, this study presents a systematic evaluation of Senegal’s mpox surveillance system within a digital public health context. Its primary aim is to assess surveillance performance, data quality, governance, and operational capacity using routine digital surveillance data and stakeholder perspectives to identify constraints to timely outbreak detection and response. The study addresses a key digital public health challenge: despite the adoption of electronic reporting platforms and intersectoral coordination mechanisms, surveillance remains largely reactive and insufficiently equipped for early warning and predictive analysis. Building on the evaluation findings, the study also examines the technical, organizational, and ethical readiness for integrating AI-enabled predictive epidemic intelligence and proposes a context-specific, policy-relevant roadmap to support a transition toward more proactive, data-driven surveillance.

The key contributions of this study highlight its novelty and practical relevance in strengthening digital public health surveillance in Senegal and beyond. They encompass system evaluation, identification of operational gaps, assessment of AI readiness, and the development of a roadmap for proactive epidemic intelligence:

- Provides the first systematic, empirical evaluation of Senegal’s mpox surveillance system within a digital public health context, integrating DHIS2 data with stakeholder insights to assess performance, governance, and operational capacity.
- Combines quantitative and qualitative analyses to reveal actionable gaps—such as reporting delays, limited interoperability, and uneven coverage—that constrain early outbreak detection and limit proactive surveillance.
- Assesses Senegal’s readiness for AI-enabled predictive epidemic intelligence, addressing governance, ethical considerations, and operational feasibility through the country’s AI4MPOX initiative.

Develops a context-specific, evidence-informed roadmap for transitioning from reactive reporting to proactive, data-driven epidemic intelligence, providing a scalable model for other African countries and Low- and Middle-Income Countries (LMICs) aligned with the Africa CDC Digital Transformation Strategy.

## Materials and methods

### Type and period of study

This study adopts a descriptive, analytical, and exploratory approach, combining quantitative and qualitative methods within a systemic, multi-source framework. This framework examines the surveillance system as an interconnected whole, considering interactions and relationships among governance, operational processes, data flows, and outputs. It also integrates information from multiple sources, such as routine DHIS2 data, laboratory records, policy documents, and stakeholder interviews, to provide a more complete and reliable understanding. This approach enables a comprehensive assessment of the system’s structure and functioning, identifying gaps and opportunities across technical, organizational, and operational domains. Considering multiple factors simultaneously allows decision-makers to develop solutions that reflect the full complexity and dynamics of the system.

The study examines mpox cases reported in Senegal from January 2024 to October 2025, a period during which the national surveillance system was enhanced. The evaluation was guided by the WHO Joint External Evaluation (JEE) and Integrated Disease Surveillance and Response (IDSR) frameworks. The JEE provides a standardized, multisectoral tool for assessing national capacities to prevent, detect, and respond to public health risks and for identifying gaps and priority actions across surveillance-related technical areas. In this study, a systematic assessment of system capacities, including real-time surveillance, reporting timeliness, multisectoral collaboration, laboratory systems, data integration, and preparedness, was conducted, with performance benchmarked against international standards. The IDSR framework, which strengthens national surveillance by integrating core and support functions across diseases and health system levels, guided the assessment of operational performance. Core functions, such as case detection timing, reporting, data analysis, laboratory linkage, and feedback, were examined alongside support functions, including workforce capacity and supervision. Together, these frameworks enabled a comprehensive evaluation of both system capacity (JEE) and functional performance (IDSR), highlighting strengths, gaps, and opportunities for improvement across the surveillance system through digital innovation.

### Study population and sampling

This study analyzes all suspected and confirmed mpox cases recorded in the DHIS2 Tracker system, validated by the Ministry of Health and Public Hygiene (MHPH) and consistent with WHO case definitions (33). A suspected case is defined as a person with an unexplained acute skin rash, mucosal lesions, or lymphadenopathy, accompanied by other symptoms such as fever, headache, muscle pain, or swollen lymph nodes, with or without an epidemiological link (e.g., contact with a confirmed case or travel to an area with mpox circulation). It also includes individuals who had contact with a probable or confirmed mpox case within 21 days prior to symptom onset and who present one of the following: acute fever (>38.5 °C), headache, myalgia, back pain, profound weakness, or fatigue. A confirmed case is a person with laboratory-confirmed mpox virus infection, as detected by real-time polymerase chain reaction (PCR) and/or sequencing of unique viral DNA sequences. For the qualitative component, semi-structured interviews (SSIs) were conducted to assess the strengths and limitations of the DHIS2 and broader surveillance system, and to explore challenges for integrating AI and modeling into epidemiological surveillance. Participants were purposively selected based on expertise in digital health, epidemiology, or mpox surveillance, including DHIS2 managers, 4S network agents, and digital health or epidemiology experts. To incorporate a One Health perspective, frontline health workers directly involved in human clinical surveillance (e.g., nurses, physicians, and community health workers) and in animal health surveillance (e.g., veterinary officers and field animal health workers) were also included. Participants were required to have direct involvement or substantial experience in surveillance operations, outbreak response, or health information management relevant to their role. This included: DHIS2 managers, 4S network agents, and digital health/epidemiology experts (surveillance operations and system management); frontline human health workers (patient care, case detection, and reporting); frontline animal health workers (animal health surveillance and outbreak reporting); members of the CVACi (Integrated Community Alert and Monitoring Committee) and community health workers (community-level case detection, reporting, or health promotion). A total of 30 interviews were conducted, approximately distributed as follows: DHIS2 managers (4), 4S network agents (4), digital health and epidemiology experts (4), frontline human health workers (6), frontline animal health workers (6), and community health workers (6). Recruitment continued until thematic saturation was reached, defined as the point at which additional interviews yielded no new insights or themes. This approach ensures the sample size was sufficient to capture diverse perspectives across stakeholder groups while maintaining methodological rigor.

Data collection used multiple sources to examine mpox case trends and explore the integration of AI and mathematical models. Epidemiological data from the DHIS2 Tracker included suspected and confirmed cases, symptoms, dates of onset, notification and confirmation status, vaccination status, and contact tracing. Sociodemographic data encompassed age, sex, occupation, mobility, and patient location, enabling analysis across population groups. Spatial data supported mapping outbreaks and analyzing regional or district trends.

The data analyzed were extracted from the DHIS2 Tracker module through manual export in Comma-Separated Values (CSV) format via the user interface, under the responsibility of the Head of the Epidemiological Surveillance and Vaccination Response Division. This approach was adopted for two main reasons: (i) the absence of secure and fully functional application programming interface (API) access, and (ii) the objective of assessing the operational quality of routinely collected surveillance data. The resulting structured datasets were regularly exported and securely transferred for integration into analytical workflows supporting epidemiological analyses, geospatial mapping, and predictive modeling. Semi-structured interviews assessed the strengths and limitations of the DHIS2 and 4S sentinel networks, highlighting needs and challenges for integrating AI and models into epidemiological surveillance. Although the findings are not generalizable, purposive sampling and triangulation improved contextual validity. This multi-source data supported a comprehensive and integrated approach to surveillance and risk mitigation.

### Data processing and analysis

Data preprocessing was conducted to prepare the dataset for analysis while preserving its value for digital surveillance system assessment. Missing values were retained and analyzed as an inherent indicator of surveillance system performance rather than treated as errors. Duplicate records were identified by cross-checking key variables, including date of symptom onset, date of notification, health district, age, and sex. When records were strictly identical, duplicates were removed following validation with the head of the surveillance division. Analyses were performed using R software to evaluate data completeness, internal consistency, and operational usability within the national surveillance system. No predefined data quality thresholds were applied, allowing for an objective characterization of the current performance of the digital surveillance system and the identification of potential biases related to data collection and data entry processes.

*Quantitative analyses* included statistical descriptions, such as calculating central tendency and dispersion measures for quantitative variables and summarizing frequencies for qualitative variables. The cumulative incidence, or attack rate, was defined as the ratio of confirmed cases to the population at risk. The case fatality rate was defined as the number of deaths per confirmed case. Case characterization included analyses of spatial, sociodemographic, and temporal distributions. Spatial distribution, mapped by region, identified areas of concentration or spread. Sociodemographic distribution was assessed by age, sex, and occupation, while temporal analysis tracked weekly and monthly trends to detect seasonal variations and periods of transmission. Assessments of delays and biases, such as the time lag between symptoms, notification, sampling, diagnosis, and reporting, were essential.

*Qualitative analyses*: All sessions were audio recorded, transcribed, anonymized, and stored securely. Thematic content analysis was performed using ATLAS.ti. A coding framework was developed deductively based on the study objectives and refined inductively through iterative review of transcripts. Triangulating data across SSIs and a literature review enabled the identification of recurring themes and differences. This analysis highlighted institutional, technological, and sociocultural barriers that could influence the effectiveness and acceptability of advanced digital tools.

*Systemic and multi-source analysis:* Finally, epidemiological, sociodemographic, spatial, and qualitative data were cross-referenced to provide an integrated view of mpox surveillance in Senegal. This approach considered the country’s specific epidemiological, socio-behavioral, and institutional characteristics, providing a robust framework for enhancing surveillance and health response planning.

### Ethical considerations

The study safeguarded patient confidentiality and anonymity by using anonymized data to protect their identities. It followed the guidelines of the National Ethics Committee (SEN23/121), ensuring adherence to ethical standards in the management of health information. For qualitative interviews, informed consent was consistently obtained, confirming voluntary participation and the proper use of data for research objectives.

## Results

### General characteristics of mpox surveillance in Senegal

#### Organization of the epidemiological surveillance system in Senegal

In line with WHO/AFRO guidelines, Senegal’s surveillance system integrates four complementary approaches:

- Passive surveillance, based on routine disease and event reporting by health facilities, laboratories, and community actors;
- Active surveillance, which involves proactive case finding through field visits, regular engagement with reporting sources, and review of clinical or hospitalization records, particularly during outbreaks or for priority diseases;
- Sentinel surveillance, conducted at selected sites to systematically collect detailed data for early outbreak detection and trend monitoring;
- And sero-epidemiological surveillance, which relies on population-based surveys to assess antibodies or biological markers, providing insights into population exposure, immunity, and transmission dynamics not captured through routine reporting.

Senegal’s epidemiological surveillance is grounded in the International Health Regulations (IHR, 2005) and follows the Integrated Disease Surveillance and Response (IDSR) framework, a unified system that integrates multiple disease reporting channels. This framework strengthens outbreak detection, reporting, and response by standardizing case definitions, streamlining data collection, and linking laboratory results to field reports, enabling faster, more accurate situational awareness. It also supports integrating One Health approaches and AI-based predictive models for proactive outbreak management. This multi-layered surveillance system is further strengthened by the Sentinel Syndromic Surveillance Network (4S), launched in 2012, which complements routine active and passive surveillance by providing real-time, nationwide monitoring of key syndromes such as fever, diarrhea, and arboviruses. The network collects data via an Android application and integrates inputs from health facilities, community and animal health workers, laboratories, and private providers. Data are validated and analyzed on an early warning platform, enabling rapid detection and response to emerging outbreaks. Nationwide tracking of syndromes enhances situational awareness, supports IDSR reporting, and lays the foundation for incorporating One Health data into predictive epidemic modeling.

The Disease and Response Information System (SIMR) is Senegal’s primary epidemiological surveillance tool. Launched in the 2000s, it adopted WHO’s IDSR approach to improve early detection and epidemic response. The goal was to unify surveillance for diseases such as cholera, measles, and malaria. In 2014, the Ministry of Health published the National SIMR Guidelines, creating a unified framework for data collection, analysis, interpretation, and sharing. The guidelines introduced Indicator-Based Surveillance (IBS), which uses routine health facility data, and Event-Based Surveillance (EBS) for the rapid detection of public health signals. That year, Senegal integrated SIMR into the DHIS2 platform for electronic data transfer from health districts, boosting system responsiveness. Through its connection to DHIS2, it ensures that disease information flows efficiently from communities to national decision-makers, enabling early detection, prompt investigation, and effective epidemic response (34).

At the local level, health posts use standardized forms to report suspected cases, supported by community workers and clinicians who follow MHPH criteria. Community Health Workers (CHWs) and the Integrated Village Committee for Community Surveillance and Alert (CVACi) collect data on diseases, deaths, illnesses, and environmental factors. Initially focused on maternal and neonatal health, CVACi expanded to include infectious diseases and zoonoses for early detection. They play a key role in Event-Based Surveillance (EBS) by serving as the first link in the alert chain at the community level. When a signal is detected, it is immediately communicated to them or the nearest health post, which then transmits it to the health district through the SIMR system. Data are entered into DHIS2 and transmitted electronically to higher levels (district, regional, national), enabling real-time access and alerts when thresholds are exceeded. At the national level, epidemiologists analyze data to identify trends, compare patterns, and detect potential outbreaks early. Diagnostic labs confirm cases, and verified alerts prompt field investigations and lab tests. Weekly reports go to the Health Emergency Operations Center (COUS) and the National Epidemic Management Committee (CNGE), which review data and coordinate the response. Regular feedback, provided through reports and dashboards, keeps all stakeholders informed and enables prompt responses.

The Réseau 4S monitors symptoms such as fever, respiratory issues, and diarrhea at select health facilities across Senegal. It is part of the national epidemiological surveillance system, providing early warning for both routine and event-based monitoring (35). Data from community surveillance units including the **CVACi** and Community Health Agents are electronically transmitted to regional and national health authorities for review. By comparing current data with historical trends, the system can identify unusual patterns that indicate potential outbreaks. The 4S network links community alerts, sentinel data, and decision-makers to enable early detection and rapid response to health threats. To enhance responses to diseases like dengue, cholera, and Ebola, Senegal launched EWARS in 2018. It gathers real-time data from health facilities, workers, and sentinel sites and generates alerts through analysis. While 4S tracks syndromic trends, EWARS integrates this data with community alerts, IBS, and EBS within the SIMR framework for coordinated action. Using DHIS2, EWARS visualizes data to facilitate timely feedback, response, and monitoring, connecting frontline surveillance with decision-makers. Alerts prompt rapid response teams to contain outbreaks.

Senegal’s epidemiological surveillance system uses a comprehensive, multi-layered approach that integrates community alerts, sentinel sites, routine reporting, and event-based monitoring through the DHIS2 platform. Complementary networks, such as EWARS, Réseau 4S, and CVACi, enhance early detection and enable rapid responses to emerging health threats. In this study, the DHIS2 and 4S sentinel networks primarily serve as passive surveillance systems, complemented by targeted active activities to strengthen case detection and support the integration of predictive modeling for outbreak monitoring. This coordinated framework aims not only to strengthen epidemic preparedness and safeguard public health but also to adopt a One Health perspective, recognizing the interconnectedness of human, animal, and environmental health. Within this system, surveillance for MPOX has been specifically structured to monitor both human and animal cases, ensuring timely detection and response to prevent potential outbreaks.

#### Organization of the mpox surveillance system in Senegal

Senegal’s Mpox surveillance system is built on a robust epidemiological foundation that integrates routine reporting, sentinel site monitoring, and event-based surveillance, enabling rapid detection and response to emerging public health threats.

In August 2024, the MHPH announced the nationwide deployment of Mpox surveillance, with targeted strengthening in high-risk areas and enhanced monitoring at critical entry points, including airports and seaports. This system integrates passive and active approaches to ensure comprehensive outbreak monitoring.

Passive surveillance relies on routine facility-based reporting through the national Health Management Information System 2 (DHIS2). All 46 health districts submit weekly reports, regardless of case detection, ensuring continuous data capture and completeness. Mpox surveillance is fully integrated into this system, with DHIS2 providing real-time situational awareness, trend analysis, and early warning. The DHIS2 Tracker extension enables case-based monitoring by linking clinical information, laboratory results, and contact tracing data, ensuring that individual case management is directly connected to national dashboards for rapid, evidence-based decision-making.

Active surveillance uses field-based interventions to proactively detect cases. Forty-six (46) health districts and ten (10) high-risk entry points were designated for intensified monitoring based on strategic location, cross-border traffic, and proximity to markets or major religious gatherings. Active screening at these sites enabled early identification of symptomatic travelers, daily reporting, and timely interventions to reduce the risk of imported cases and onward transmission. Field teams conducted investigations, contact tracing, and community-level case finding to complement routine facility-based reporting, improving detection completeness and supporting rapid outbreak response. During mass gatherings, such as the Magal of Touba pilgrimage in August 2024, active surveillance was further intensified. A total of 186 dedicated surveillance sites were established for pilgrims, testing and quarantine facilities were activated, and health staff received additional training to ensure rapid detection and containment of cases. This surge capacity demonstrated the system’s flexibility in adapting to large-scale public health events while maintaining both routine and targeted surveillance efforts. In August 2025, a case involving a foreign national triggered activation of the surveillance and response system. This included isolation, contact monitoring, and multisectoral coordination. The Mpox emergency response framework included key components to ensure prompt detection and action. At its core was activation of a national incident management structure led by the COUS. This body oversees nationwide preparedness and response efforts, including case detection, reporting, laboratory confirmation, risk communication, community engagement, and coordination. Additionally, the system promoted close collaboration with national reference laboratories, such as the National Laboratory for Livestock and Veterinary Research and the Institut Pasteur de Dakar, to facilitate swift pathogen confirmation and genomic surveillance.

Senegal’s strategy relies on a comprehensive One Health framework that links human, animal, and environmental health. It promotes cooperation among ministries (Health, Livestock, Environment) and is managed by a National One Health Platform. Community initiatives have trained health workers, veterinarians, and environmental officers to enhance local surveillance of zoonotic diseases. At the strategic level, the High National Council for Global Health Security “One Health” (HCNSSM–OH), created in 2017, guides One Health and global health security, overseeing cross-sector governance, resources, and coordination to ensure collaboration among sectors related to health, food security, safety, and security. Unlike disease-specific programs, HCNSSM–OH sets strategic priorities and guides Senegal’s One Health efforts and epidemic preparedness.

Overall, Senegal’s Mpox surveillance integrates with national health information systems, enabling rapid response through COUS and a multisectoral One Health approach that connects human, animal, and environmental health. These elements enhance early detection, response, and containment, laying the groundwork for analyzing Mpox cases and trends.

### Quantitative analysis of suspected and confirmed cases of mpox

**Figure 1:**
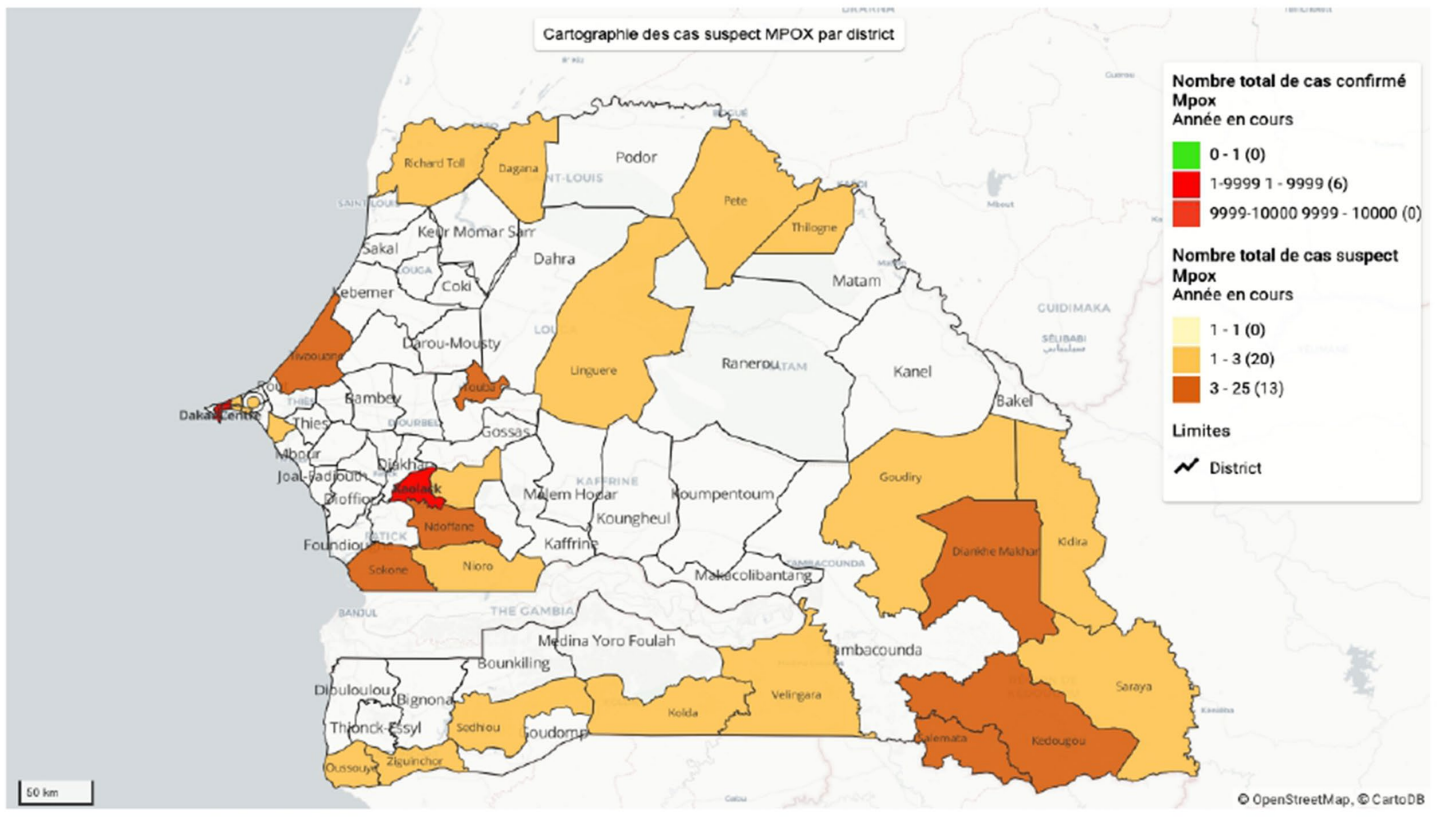
Map showing the distribution of suspected and confirmed mpox cases by region.

Building on the national Mpox surveillance system’s structural and operational foundation, a quantitative analysis of reported cases provides valuable insights into epidemiological trends and the effectiveness of current interventions. Since the WHO declared mpox a public health emergency of international concern, Senegal has enhanced its epidemiological surveillance efforts to prevent the disease’s entry and spread. By October 31, 2025, Senegal had reported a total of 277 suspected Mpox cases, of which 7 were laboratory-confirmed and 2 were classified as probable. Clinical data were available for 136 suspected and confirmed cases analyzed during this period. Confirmed cases remain geographically concentrated (Figure 1), with all 7 cases identified in Dakar, yielding a confirmation rate of 4.5%.

Among confirmed cases, the majority (four individuals) have recovered, while two continue to receive treatment, reflecting the effectiveness of case management and clinical follow-up. The presence of two probable cases, one IgM-positive and one IgG-positive, indicates recent and past exposure within the population. Overall, these data indicate that although the absolute number of confirmed Mpox cases remains limited, the surveillance system is actively capturing suspected cases nationwide.

#### Epidemiological and clinical profile of Mpox in Senegal

**Figure 2:**
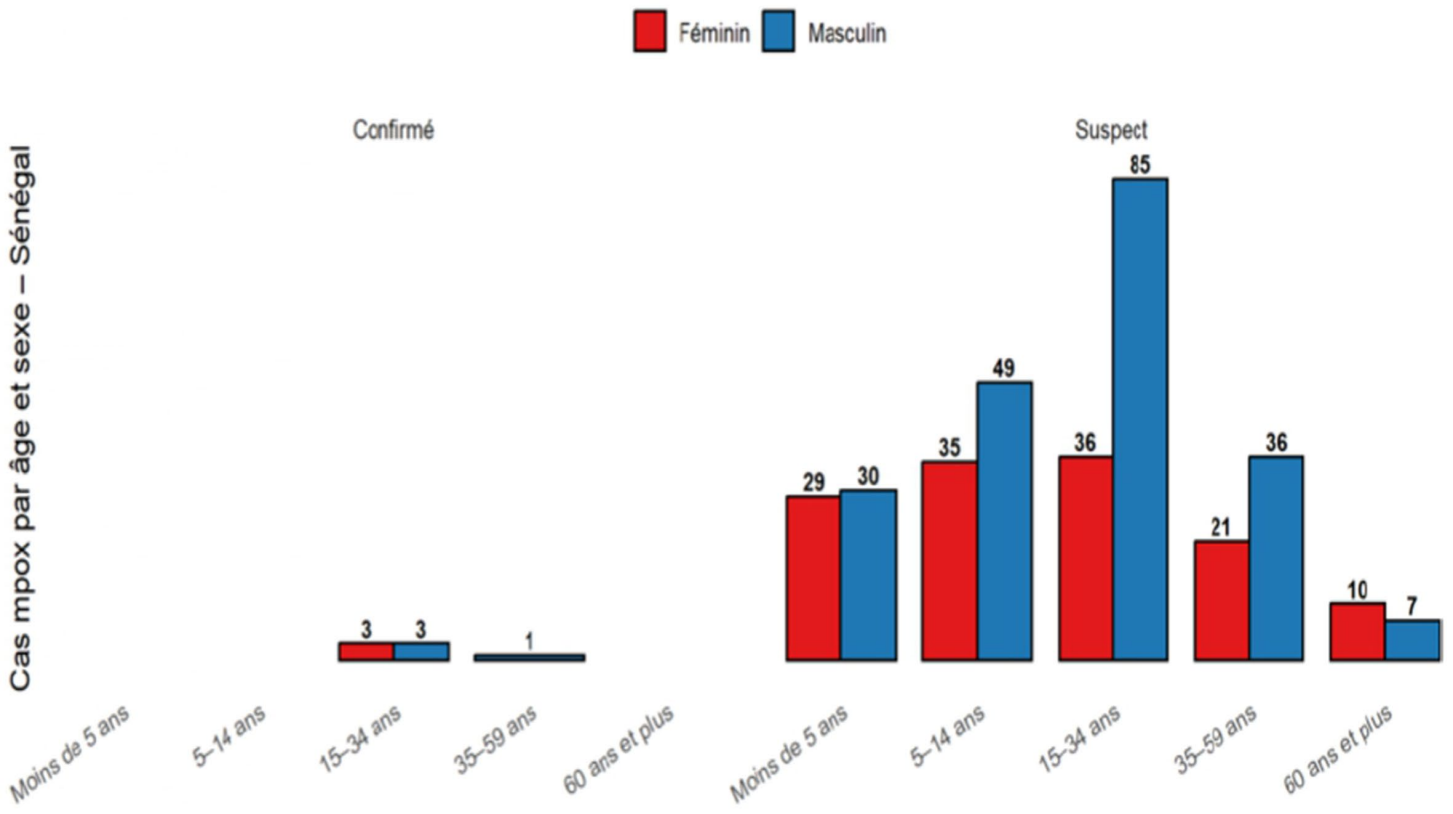
Distribution of suspected mpox cases by gender and age group.

Analysis of Mpox surveillance data in Senegal indicates that the average age of suspected and confirmed cases is 21.4 years (±18.2), with a wide age range from 1 to 95 years. The predominance of young adults aged 20–40 years—especially males—among both suspected and confirmed cases (Figure 2) mirrors trends observed in Ghana and other West African countries, where the 20–29 age group has been the most affected (36).

The predominance of male cases underscores gender differences in Mpox exposure and susceptibility, largely influenced by occupational and behavioral factors such as outdoor activities, travel, and animal contact. It may also reflect variations in healthcare-seeking behavior and risk perception. Although behavioral and social factors likely affect transmission patterns, their precise roles remain unclear. The age distribution suggests that transmission is correlated with mobility, social interactions, and employment, particularly among young adults aged 20–40, who are more active and economically engaged and often participate in activities involving animals. Surveys among health workers and community members highlight behavioral issues such as self-medication, delayed treatment, stigma-related reluctance to report symptoms, and low awareness in rural settings. Continued contact with animals underscores ongoing zoonotic risks at the human–animal interface.

The vaccination history of affected individuals reveals patterns of susceptibility. Most cases occur among individuals who have not received smallpox or Mpox vaccines, reflecting the discontinuation of routine smallpox vaccination in Senegal in 1981 (37). This absence of immunological protection corresponds to the majority of cases occurring in individuals born after this cutoff, emphasizing how waning herd immunity influences current Mpox outbreaks. Smallpox vaccination is estimated to offer 80–85% cross-protection against Mpox, underscoring the benefits of past vaccination efforts and the increased risk following their cessation. In Senegal and West Africa, declining herd immunity particularly affects young adults and adolescents (38), especially those with higher mobility, wildlife-related occupations, or social behaviors that facilitate transmission.

**Figure 3:**
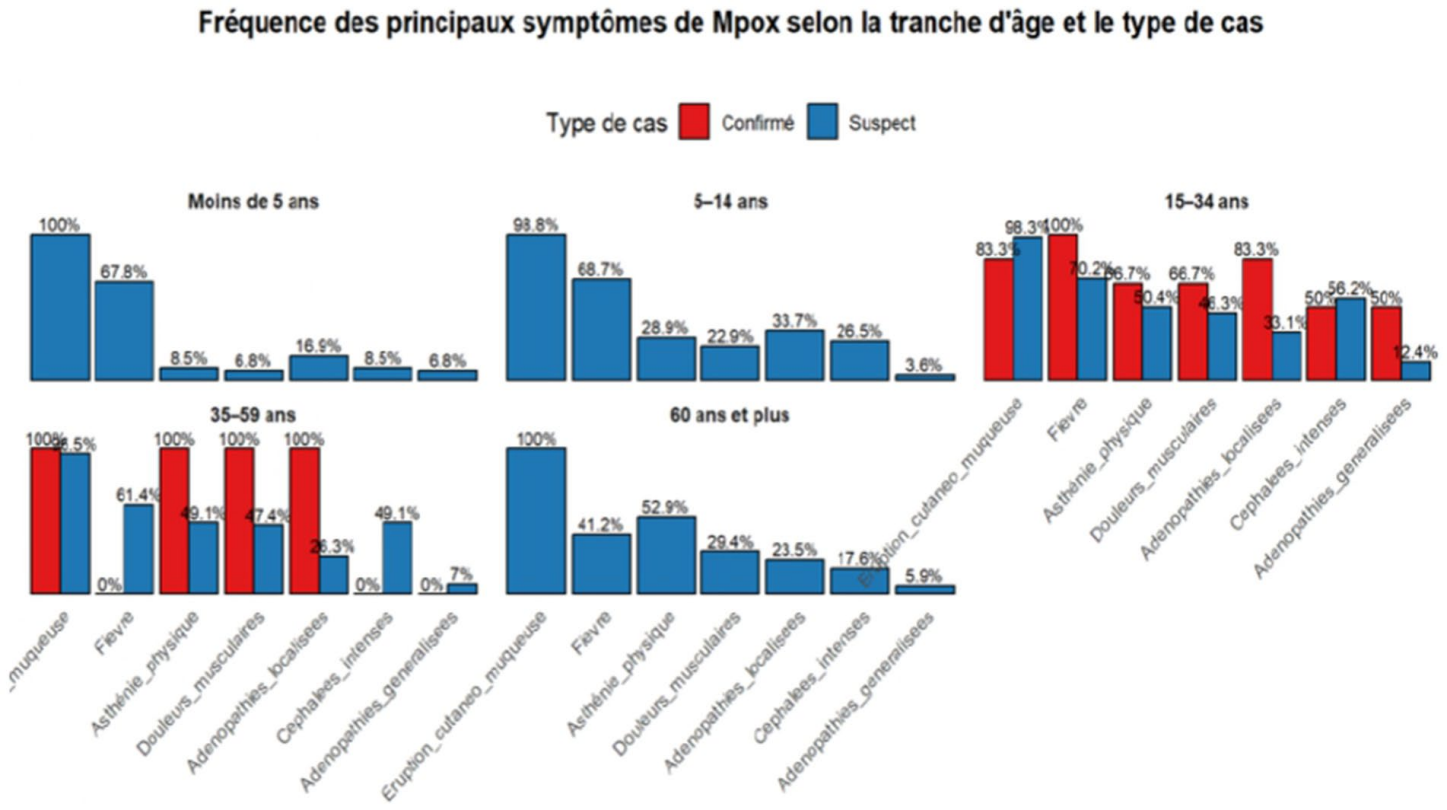
Distribution of symptom frequency in mpox cases by age and case type.

Clinically, the first imported case, reported on August 22, 2025, involved a 28-year-old foreign national who developed symptoms before arriving in Senegal. Subsequent detection of a native case of clade IIb on September 22, 2025 (distinct from the imported clade Ib) confirmed community transmission in Dakar, likely influenced by urban density and centralized diagnostic capacity. No cases were invalidated, indicating a 0% false-positive rate. The small number of confirmed cases relative to suspects may reflect heightened surveillance and the similarity of Mpox to chickenpox or shingles, which can lead to overreporting. Confirmed and suspected cases primarily presented with skin and mucosal rashes, solid lesions, fever, and headaches. These symptoms were similar across age groups but more common in males, especially those aged 5–14 and over 60 (Figure 3).

The figure shows that cutaneous and mucosal eruptions are the most consistent symptoms across all age groups, occurring in approximately 100% of confirmed cases. Fever and asthenia are also frequent, particularly among individuals aged 5–34 years, whereas lymphadenopathy and intense headaches are more common in adults aged 35–59 years. Children under 5 years old typically present with rash and fever, whereas older adults (≥60 years) exhibit fatigue and fever, accompanied by a less severe rash, likely due to immune aging or comorbidities. Unlike Central African reports (39), where more severe cases affected children and pregnant women, no gender differences are observed here. Overall, the clinical presentation varies across age groups, with skin lesions, fever, and weakness being key for detection. The overlap between suspected and confirmed cases suggests that Senegal’s case definitions accurately reflect clinical patterns, aiding early detection and isolation.

The epidemiological and clinical profile of the seven confirmed mpox cases in Senegal highlights a marked concentration among young adults aged 20–35, with infection linked to specific sexual behaviors. The predominance of unprotected sex (5 cases), including multiple partners in one case, points to sexual transmission as a key driver in this cluster (40–42). The documented presence of the virus in semen and genital secretions reinforces the biological plausibility of sexual transmission, a mode increasingly recognized in recent outbreaks beyond traditional zoonotic pathways. This pattern mirrors trends observed in other regions of West and Central Africa, including the DRC (43–45), Ghana, and Guinea, suggesting a shift in the epidemiology of Mpox from primarily animal-to-human transmission toward sustained human-to-human transmission through sexual networks, particularly among men who have sex with men (MSM) and mobile young adults (46).

Laboratory investigations have shown that varicella-zoster virus (VZV) is the most common pathogen in suspected cases, followed by human herpesvirus 7, herpes simplex virus 1, and measles. The presence of multiple pathogens underscores the diagnostic challenges posed by vesiculopustular skin eruptions in Senegal. High VZV prevalence, especially among males, suggests that many suspected mpox cases may be caused by varicella or herpesviruses, which share symptoms such as fever, rash, and vesicular lesions. Although less common, HHV-7, HSV-1, and measles can also cause rashes, and symptom-based diagnosis is limited. These findings underscore the importance of differential diagnosis, given the clinical similarities between mpox and other viral skin infections.

These findings collectively show that Senegal’s Mpox epidemiology is shaped by the intersection of age, sex, and immune history, with young, unvaccinated males being the most vulnerable group. This profile lays the groundwork for the upcoming spatiotemporal analysis of suspected and confirmed Mpox cases, which will examine regional distribution, temporal patterns, and clusters.

#### Temporal and spatiotemporal analysis of mpox surveillance in Senegal

**Figure 4:**
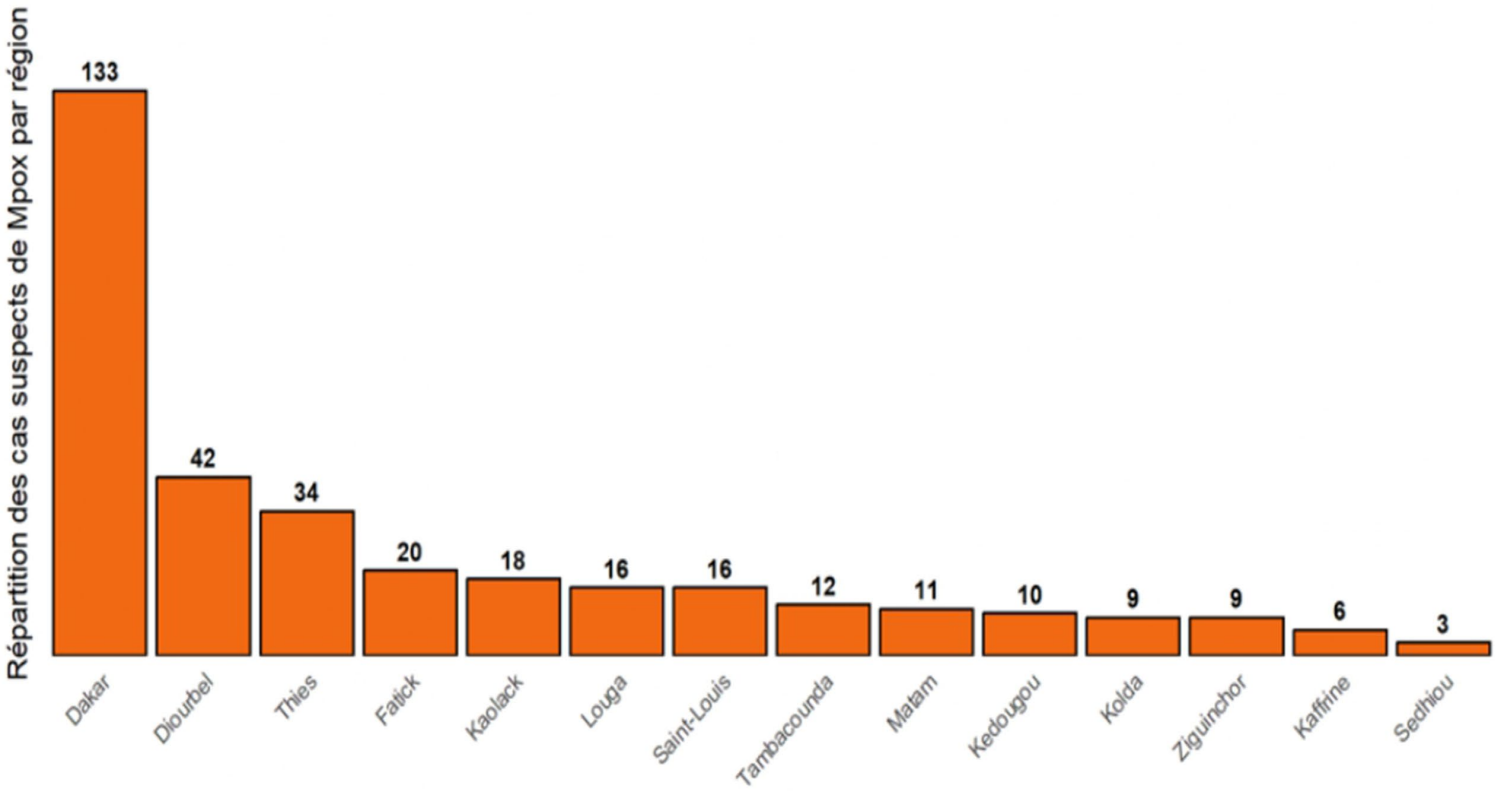
Distribution of suspected mpox cases by region.

Dakar, the capital of Senegal, serves as the country’s economic and administrative hub, accounting for the highest number of suspected Mpox cases (40.5%). Diourbel (12.2%) and Thiès (9.6%) are notable hotspots (Figure 4) because of their strategic locations along transit routes and as entry or exit points. The higher case numbers in these regions may reflect their connectivity, underscoring the need for increased surveillance to prevent spread. Other areas, such as Fatick, Kaolack, Louga, Saint-Louis, and Tambacounda, report moderate numbers of suspected cases, indicating that surveillance extends to rural and border zones. In contrast, the lower numbers in Kaffrine and Sédhiou may reflect smaller populations, limited connectivity, or underreporting.

A geographic analysis shows that suspected cases are concentrated in densely populated areas with centralized diagnostic services and active urban movement, which facilitate disease detection and transmission. Dakar, the political and economic hub, has a mobile population and better healthcare, enabling quicker case detection. Diourbel, influenced by Touba’s growth, has high density and increased religious travel, raising the risk of disease spread. Along with Thies, Diourbel serves as a transit hub along busy trade routes, which are essential for cross-border surveillance. The study finds a significant correlation between mobility, urban density, and mpox alerts (r ≈ 0.62; *p* < 0.05). Urban centers, transit regions, traders, and social contacts drive the ongoing spread. The Dakar–Thiès–Diourbel corridor, with high density and many young adults, points to widespread social and sexual networks among at-risk groups including people aged 20–40, men who have sex with men, and highly mobile individuals. Multiple suspected cases suggest targeted efforts in high-risk zones, though regional differences may stem from underreporting or limited capacity outside Dakar.

**Figure 5:**
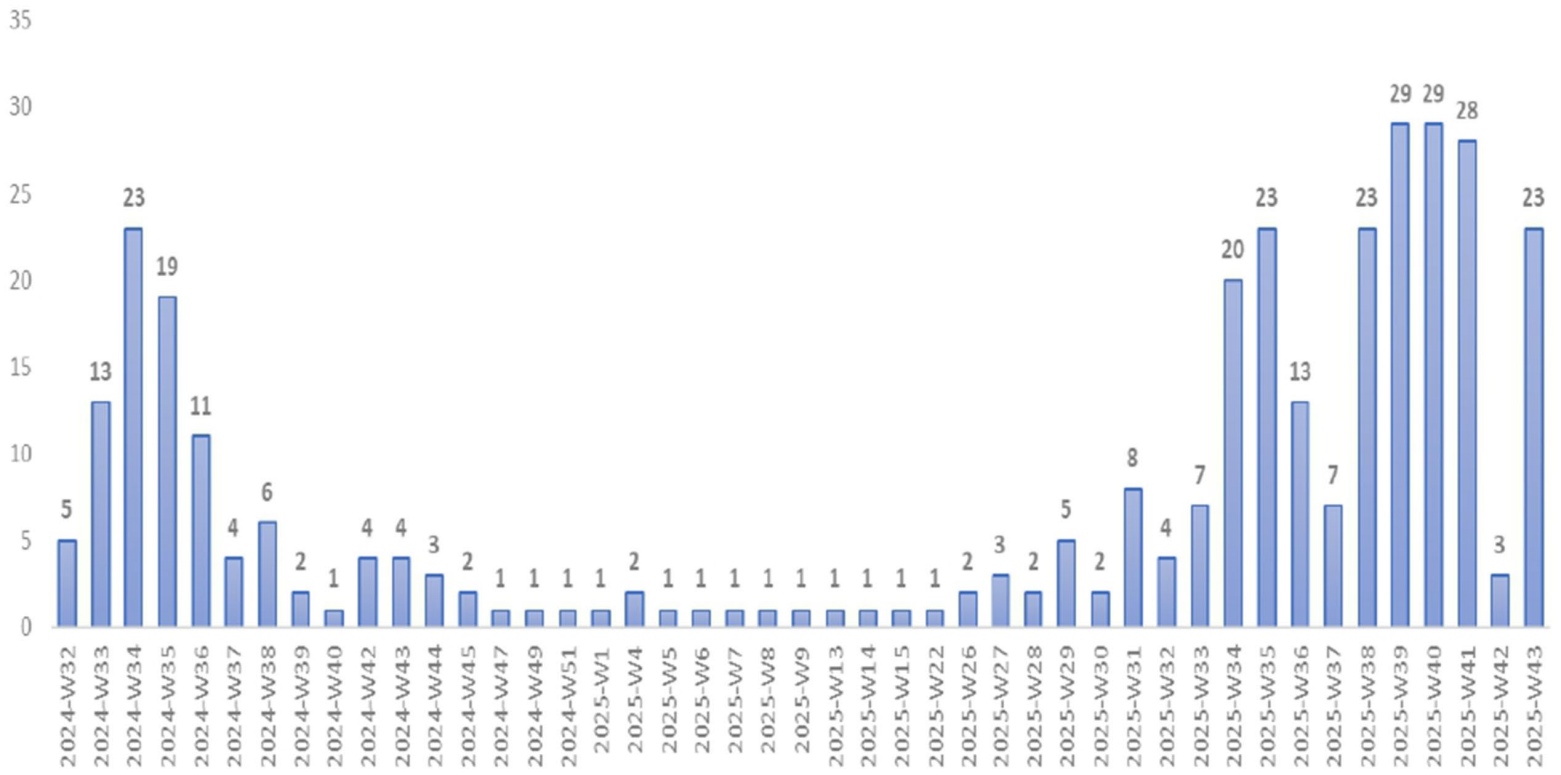
Weekly trend in suspected mpox cases, Senegal, January 2024–October 2025.

Over nearly 2 years, from week 32 of 2024 (early August) to week 43 of 2025 (late October), suspected mpox cases (Figure 5) show an initial peak in 2024, with a high of 23 cases in week 34 (late August). Following this, cases gradually decrease and remain low from late 2024 through mid-2025. Several reports over the past few weeks indicate few or no cases, reflecting a period of relative epidemiological stability. However, starting in week 36 of 2025 (early September), an evident resurgence emerges, followed by a second, more significant peak between weeks 38 and 42 (late September–early October), reaching 29 suspected cases in weeks 39 and 40 (early October). This increase may suggest a new wave of transmission or higher case detection and reporting. The decline after week 42 suggests the wave could be subsiding.

Peaks in suspected cases are linked to regional travel, seasonal trends, and government oversight. The first peak occurred in August 2024, following an increase in surveillance after a WHO alert. Subsequent surges often occur after health warnings, especially when cases appear in neighboring countries. Guinea reported its first case in September 2024, and transmission rose from June 2025 and nearly reaching 1,000 confirmed cases by the end of 2025. This prompted increased monitoring in nearby nations. In Senegal, each WHO alert triggered more administrative alerts and notifications within the DHIS2 system, particularly in border areas and regions with high population movement. These peaks coincide with vigilance notices encouraging active detection, indicating ongoing transmission mainly driven by Guinea. Alert notifications from April to June 2025 indicate an increase in cross-border activity involving Senegal, Mali, and Guinea, driven by seasonal fluctuations in animal movement and agricultural trade at the borders. For example, during the dry season—usually from February to June—roads become more accessible, boosting trade in cereals, livestock, and food across borders. Additionally, before major holidays like Eid al-Adha in July and August, significant livestock migration occurs among Mali, Senegal, and Guinea. The notifications surged again in early September 2025, after the Magal de Touba and the confirmation of Senegal’s first mpox case. This surge shows heightened awareness and surveillance efforts. Although large gatherings like the Magal increase population density and interaction—potentially aiding transmission—the first case was confirmed a month after the event. Therefore, a case appearing later is not necessarily linked to mass spread during the event. Events such as Tabaski, late May to June festivals, and Magal carry a high transmission risk through close contact, especially considering silent spread and complex chains, given the limited data available. The second case in a Senegalese resident, after the initial importation, suggests local transmission or an unknown source. This means that the absence of a direct link between an imported case and gatherings should not dismiss the role of mass events in spreading. Vigilant monitoring of transmission chains remains essential, even in the absence of a clear connection to specific events.

Overall, these quantitative results lay the groundwork for a thorough evaluation of Senegal’s Mpox epidemiological surveillance system, assessing its effectiveness, identifying potential gaps, and determining its ability to detect and respond to both imported and locally transmitted cases.

### Evaluation of the performance of the mpox epidemiological surveillance system

The performance of Senegal’s Mpox epidemiological surveillance system can be assessed by examining key attributes that define a practical public health surveillance framework: efficiency, sensitivity, completeness, timeliness, data quality, cross-sector integration, and usefulness.

1. The *effectiveness of Senegal’s Mpox surveillance system* can be evaluated using key metrics such as case detection rates, reporting delays, and laboratory confirmation times. During the study, 136 of 277 suspected cases were confirmed, including 7 laboratory-confirmed cases, for a 4.5% confirmation rate. This suggests the system effectively detects suspected cases nationwide, but the low confirmation rate indicates many suspected cases are other rash illnesses, mainly varicella-zoster virus, which accounts for about 32%. Reporting and notification delays show a median symptom-to-consultation time of about 65 days (±9.29), while the median from consultation to confirmation is 3.96 days (±5.31). In Dakar, suspected cases are reported within 1–2 days of symptom onset, in line with international standards. However, delays of 4–7 days occur in peripheral districts due to logistics, staffing, and communication issues, limiting timely responses and possibly underestimating cases outside Dakar, highlighting regional disparities. All confirmed cases were in Dakar, indicating centralized diagnostic capacity and potential under-detection elsewhere. The alert validation process, which involves district verification, triage, and coordination, takes 24–48 h, with PCR results typically available within 48–72 h in Dakar. Remote regions may experience an extra 1–2 days delay, leading to confirmation 4–5 days post-detection. While these timeframes are acceptable, they may still impede rapid responses in peripheral areas. Overall, the surveillance system is strong in urban centers but less sensitive and responsive in rural and border districts, underscoring the need for better diagnostic access and streamlined reporting outside Dakar.
2. *Surveillance coverage and sensitivity:* From January 2024 to October 2025, the DHIS2 system collected data from all Senegal regions, including urban, peri-urban, and rural areas. Coverage was comprehensive, with most health facilities reporting suspected cases. Analysis shows that 81% of notifications came from public health facilities via the DHIS2 Tracker, with the private sector, mainly clinics and labs in Dakar, contributing 8%. Syndromic detection accounted for 7%, aiding early detection, while community surveillance accounted for only 4%, limited by gaps in training, tools, and supervision. This highlights the public sector’s vital role in swiftly identifying cases, though rural areas faced delays due to limited access and awareness. Routine reporting, sentinel sites, event-based surveillance, and DHIS2 tracking support early detection, especially in high-risk zones and entry points.

Senegal confirmed at least 7 mpox cases in Dakar in 2025, including 2 probable cases identified through contact tracing. The detection of an imported case in August 2025 and subsequent local transmission highlight the system’s operational sensitivity. Despite a swift response to the initial case in August 2025, detection is slower in rural and border areas, which can delay identification. Sensitivity, estimated at 50–80% (median 65%), suggests that about one-third of cases may go unnoticed due to centralized diagnostics, limited rural coverage, unawareness of atypical symptoms, and stigma-related fears among health professionals.

3. *Data quality* in notification forms and DHIS2 is evaluated based on accuracy, consistency, and completeness. Senegal’s epidemiological surveillance benefits from standardized case definitions, laboratory confirmation through institutions such as the Institut Pasteur de Dakar, and the linking of clinical, laboratory, and contact-tracing data in DHIS2 Tracker to ensure accurate information. Completeness is good, with over 95% of fields—such as demographics, symptoms, location, and contacts—filled, and key variables like age, sex, residence, vaccination, and exposure exceeding 85% completion. However, 15–20% of animal contact and travel data are missing or incomplete, affecting risk analysis. Data are consistent, with symptoms aligning with those of mpox, and frequent blood and skin testing is recommended. Internal audits indicate that most Dakar data are accurate, with error rates below 5%. Some remote regions show inconsistencies that affect epidemiology. Unique identifiers help reduce duplicates, though delays in data updates can suggest incompleteness.
4. *Community engagement* is a vital yet complex element of Senegal’s Mpox surveillance, influencing how quickly and thoroughly cases are identified Community members usually do not report suspected Mpox cases directly to health authorities. Instead, they must first notify a CHW, who verifies the report, completes standard forms, and forwards it to the health post or district focal point. Although this hierarchical process helps maintain data accuracy and proper reporting, it can cause delays, especially in regions where CHWs serve large populations or lack logistical support. Despite regular training and established strategies, effectiveness varies regionally. In rural and border districts, low CHW density and transportation challenges can delay alerts from remote villages by days. Mistrust, stigma, low awareness, and fatigue among CHWs further hinder timely reporting. Community initiatives involving teachers, religious leaders, farmers, and traders have increased awareness and vigilance; however, the absence of direct alert channels at the community level limits the effectiveness of early warnings. Enhancing surveillance requires enabling community members to report directly via simplified or digital systems, increasing CHW coverage, and incentivizing prompt communication within the community–health system link. Overall, though community engagement is crucial, the current indirect reporting process remains a significant obstacle to early detection and swift response.
5. *Cross-sectoral coordination*, anchored in the One Health framework, is central to Senegal’s strategy for managing zoonotic threats such as Mpox. Effective epidemic intelligence, particularly for zoonotic diseases, cannot be achieved through isolated sectoral efforts; it requires a unified surveillance system that can detect early signals across interconnected domains (47). In the Senegalese context, livestock movement, animal trade, and ecological factors play a crucial role in shaping zoonotic transmission dynamics, underscoring the need to link human health surveillance with veterinary and environmental monitoring systems.

Despite well-established coordination mechanisms, operationalizing cross-sector collaboration in Mpox surveillance remains limited. Each of these structures plays an essential role: the CNGE oversees epidemic preparedness and inter-ministerial coordination; the COUS ensures real-time emergency management and rapid response; and the HCNSSM–OH provides strategic leadership in aligning human, animal, and environmental health policies within the One Health framework. However, despite these complementary mandates, data integration and information flow across sectors remain fragmented. Health, veterinary, and environmental surveillance systems operate primarily in parallel, with limited interoperability and inconsistent data-sharing protocols. This structural disconnect constrains real-time situational awareness, delays risk assessment, and weakens collective decision-making during outbreaks. As a result, while institutional coordination mechanisms exist, the practical implementation of a unified One Health surveillance approach in Senegal remains in development, requiring stronger cross-sector communication, shared digital infrastructure, and joint analytical frameworks to achieve full integration.

Creating an integrated surveillance system that links veterinary data, human reports, and environmental indicators would improve early warning, transparency, and evidence-based decision-making. Transitioning from reactive to proactive, intelligence-driven surveillance is crucial for Senegal to prevent zoonotic threats, such as Mpox, and to incorporate AI and modeling into epidemic intelligence. Improving One Health requires developing standardized digital tools, enhancing data sharing, and expanding the use of AI for cross-sector analysis. These technologies can improve prediction accuracy, optimize resource use, and enable real-time decision-making within Senegal’s evolving One Health framework.

### Prospects for integrating AI and mathematical modeling within mpox surveillance

#### Identified needs and future directions for strengthening mpox surveillance in Senegal

The 2025 evaluation of Senegal’s Mpox surveillance system identified structural and operational issues, including low community involvement and fragmented information-sharing, that hindered its effectiveness. Addressing these issues is vital for better detection, rapid response, and a One Health approach. Three main areas for improvement are identified.

1. *Responsiveness and Community Engagement*: To improve responsiveness, automating data collection from field reports, mobile apps, and surveys is essential to reduce delays and errors. Offline mobile tools enable data transfer in areas with limited connectivity, ensuring continuous reporting. Strengthening community engagement is vital for Senegal’s Mpox surveillance by working closely with CHWs, local leaders, and community groups to expand coverage and detect cases early. Providing digital tools or alert systems, such as mobile apps or SMS, enables community members to report suspected cases directly, even in areas with limited connectivity. This reduces reliance on intermediaries, shortens response times, and improves the timeliness and sensitivity of the surveillance system.
2. *Intersectoral Data Integration, Analytics, and Decision-Making:* Effective Mpox control depends on timely, coordinated decisions across human, animal, and environmental health sectors. Incorporating artificial intelligence (AI) and predictive analytics into this multisectoral system would enable real-time epidemiological analysis, enhance alert accuracy, and expedite laboratory confirmation. Shared data platforms linking DHIS2 to veterinary and environmental databases would enable cluster detection, disease-spread forecasting, and the prioritization of interventions.
3. *Mathematical Modeling and Resource Optimization:* Mathematical models can simulate Mpox transmission dynamics, assess the effectiveness of interventions such as isolation, vaccination, and social distancing, and estimate the risks of inter-regional spread. These tools help identify high-risk areas, predict outbreak trends, and plan scenarios to optimize resource utilization. Incorporating modeling into surveillance activities enhances response adaptability and efficiency, especially where resources are limited.

Integrating AI with mathematical modeling offers an opportunity to enhance Senegal’s Mpox surveillance by making it more predictive and data-driven. AI analytics can enhance early warning systems by detecting anomalies, forecasting outbreaks, and identifying hotspots, using data from human cases, animals, the environment, and movement patterns. A national epidemic platform that integrates DHIS2 data with veterinary, weather, and mobility information would enable quicker, more targeted responses, thereby increasing the overall effectiveness of the surveillance system. This platform also forms the foundation for our proposed project to apply predictive analytics and cross-sectoral data sharing for mpox surveillance.

#### AI4MPOX-SN: towards intelligent and predictive surveillance of Mpox

The AI4MPOX-SN initiative addresses current surveillance challenges, including reporting delays, scattered intersectoral data, and limited community engagement. It aims to leverage AI and mathematical modeling to enhance early detection, predict outbreaks, and support decision-making across the human, animal, and environmental health sectors. Emphasizing a One Health–oriented, predictive, and evidence-based strategy, the project combines technological innovations with community engagement and multisectoral collaboration. Its goal is to transform Mpox surveillance from a reactive to a proactive, intelligence-driven system that can forecast and mitigate outbreaks across Senegal. Key components include three modules:

*- A comprehensive digital platform* will form the backbone of the national Mpox surveillance system. It serves as a central hub for integrating and distributing data from sources such as DHIS2 Tracker, the 4S network, laboratories, sentinel sites, peripheral structures, and data on human, animal, and environmental mobility. Designed to enhance epidemic intelligence through a One Health approach, the platform features: (1) Data collection via API connectors, community mobile forms, apps, SMS, and social networks; (2) Data cleaning and standardization to ensure quality; (3) Interoperability across health platforms for coordinated sharing and real-time anomaly detection; (4) Hosting analytical models, including AI and mathematical algorithms; (5) Interactive dashboards with key indicators, risk maps, outbreak data, and forecasts. A gamified feedback system with supervised learning will enhance data quality, providing validators with notifications for investigations. Overall, it aims to detect early signals, enable swift responses, and motivate stakeholders through the use of AI tools, supporting the digital surveillance transformation.

*- AI, a catalyst for Analysis and Forecasting*: AI improves predictive analysis, early detection, and decision support. It processes large datasets of epidemiological, environmental, and social data to support planning and response efforts. AI operates at various levels within surveillance systems (Table 1).

**Table 1:** Levels and techniques of AI application for the detection and management of mpox in Senegal.

Level of application	Main objective	Techniques/algorithms used
Early detection	Identify weak signals that indicate an outbreak	Random forests, SVM, logistic regression
Spatio-temporal analysis	Detect emerging clusters from georeferenced data	K-means, DBSCAN, spatio-temporal models
Epidemiological prediction	Anticipating the spread and intensity of cases	Recurrent neural networks (LSTM), temporal CNNs
Resource optimization	Prioritizing areas for intervention and allocation	Decision models, supervised learning
Data quality analysis	Detect inconsistencies or outliers	Autoencoders, anomaly detection
Generative AI (RAG)	Raise awareness and provide training via an educational chatbot	Generative model backed by an internal document database

This module integrates three main analytical tools for epidemiological forecasting: *nowcasting* estimates unreported cases in real time by using regression algorithms or recurrent neural networks to detect early epidemic signals; *forecasting* offers short- and medium-term projections (1 to 4 weeks) for cases, hospitalizations, and healthcare resource needs, aiding operational planning and intervention prioritization; and *cluster detection* uses AI and geostatistical methods to identify potential outbreaks before clinical confirmation, guiding field investigations and response efforts. It also features a generative AI chatbot built on a Retrieval-Augmented Generation (RAG) architecture. By merging a semantic search engine with a language model, it retrieves relevant data from a verified internal database to generate informative responses. Integrated into the AI4MPOX-SN platform, it serves as an educational tool to raise awareness and provide training, answering questions from the public and healthcare workers about Mpox. It utilizes sources such as national protocols, WHO guidelines, and educational materials. Its primary aim is to improve community awareness through accurate information and support ongoing health professional training with tailored responses. By enhancing health literacy, encouraging participation, and improving access to information, the RAG chatbot helps strengthen the health system’s resilience against Mpox.

*Mathematical modeling* helps understand, simulate, and plan by revealing transmission mechanisms and forecasting intervention effects. While AI detects and predicts based on data, modeling provides explanatory insights vital for health planning. This approach uses various models: (1) Compartmental models (such as SEIR) simulate disease spread and estimate transmission; (2) stochastic models incorporate uncertainty through randomness; (3) spatio-temporal models incorporate mobility and density to forecast spread; (4) multi-agent models simulate behaviors for targeted interventions and scenario testing. Many models lack integration of social and cultural factors, suffer from reporting issues, and underrepresent low- and middle-income countries, often failing to stratify by vulnerability (48). For Senegal, models should incorporate social vulnerabilities, including age, comorbidities, socioeconomic status, occupation, mobility, and access to healthcare, using census and survey data. These factors should extend beyond demographics to encompass structural, behavioral, and contextual elements (49). Population groups and individual attributes should be based on these factors, with input from social scientists and health experts, to accurately capture social heterogeneity.

The system’s architecture, based on an integrated structure that connects collection, analysis, and decision-making (Table 2), ensures a continuous flow of information among the platform, AI modules, modeling, and tools such as dashboards and a RAG chatbot. This closed-loop system enables adaptive, predictive surveillance, with data, analysis, and alerts feeding into one another.

**Table 2:** Architecture of the AI+modeling system for epidemiological surveillance of mpox in Senegal.

Level	Component	Main function
Collection	AI4MPOX-SN platform	Data centralization and quality
Analysis	AI + Modeling	Detection, prediction, and simulation
Distribution	Dashboards + RAG chatbot	Communication, training, awareness

#### Identification of relevant variables for modeling

The success of integrating AI with mathematical modeling in mpox surveillance depends on selecting high-quality, relevant variables for the predictive models. For accurate mpox transmission modeling in Senegal, it is essential to select variables that capture epidemiological trends, human behavior, geography, and environmental factors unique to Senegal (Table 3).

**Table 3:** Local variables relevant for modeling.

Category	Specific variables	Usefulness for modeling
Epidemiological	Confirmed cases, suspected cases, notification delays, identified clusters, fatality rates, number of human-human/animal-human contacts traced	Allows calibration of transmission dynamics and identification of emerging outbreaks.
Demographic	Population density, distribution by age and gender, occupation, household structure, urbanization rate, informal housing	Influences the spread of the virus and at-risk groups.
Mobility/human and animal flows	Interregional travel, seasonal migration, cross-border flows, animal transport and trade, market attendance	Enables modeling of the spatial spread of the virus and anticipation of risk areas.
Socio-behavioral	Livestock farming practices, frequency of human-animal contact, dietary habits, use of formal/traditional healthcare, risk perception, community engagement; local knowledge of transmission, reservoirs, risk areas	Affects early detection, case reporting, and the effectiveness of interventions.
Environmental	Proximity to forest areas, climate, land use, presence and density of animal reservoirs (rodents)	Factors promoting the emergence and spread of zoonoses.
Health systems/surveillance	Local health coverage, accessibility of health centers, reporting efficiency, diagnosis time	Influences the speed of notification and the quality of data collected.
Technological/Digital data	GIS data, DHIS2 Tracker, 4S network, mobile telephony, social networks; Language of data collection and dissemination	Enables real-time feed integration for AI and predictive detection.
Governance and coordination	Existence of local emergency plans, frequency of coordination meetings, level of health decentralization	Their inclusion helps explain interregional variability in surveillance effectiveness and response speed.

All variables are integrated into models that use temporal, spatial, and behavioral data to calibrate transmission rates, forecast spread, and identify at-risk populations. Incorporating socio-behavioral and ecological factors, often missing from standard methods, is crucial for understanding transmission and tailoring models for Senegal. Historical mpox data helps project future scenarios, considering delays and mobility, to pinpoint high-risk areas, link past trends to predictions, improve risk assessment, and inform resource allocation. These tools support decision-making and outbreak preparedness, and machine learning enables adaptive, participatory, and interoperable surveillance systems.

#### Ethical framework and AI governance

Given the widespread use of sensitive health, behavioral, and mobility data, it is crucial to establish robust ethical frameworks and AI governance structures. These should ensure the responsible, transparent, and fair deployment of technology. AI explainability and thorough documentation of algorithms, including type, version, sources, performance, and limitations, improve accountability and reproducibility. Human oversight ensures that decision-making remains centered on humans.

*Health data is sensitive. If AI is implemented without a clear framework, people may lose confidence in the system... We must ensure that AI does not replace humans. A public health expert must always validate analysis. digital health expert, in charge of system management*

The quote underscores the importance of strengthening governance, transparency, and community engagement from the outset. AI in mpox surveillance should be introduced gradually, inclusively, and ethically, combining human expertise with interoperability while minimizing bias. Engaging stakeholders—including community members, health officials, and experts—is crucial for creating frameworks that align with ethical standards and local norms, fostering trust, community engagement, and social acceptance. The AI4MPOX-SN project, governed fairly and inclusively, promotes contextually relevant, morally grounded, and decolonial AI that meets international standards and aligns with Senegal’s priorities. It values local knowledge, maintains independence and data sovereignty, and aims to develop trustworthy local AI to strengthen health resilience across Africa.

## Discussion

*Key lessons from Senegal’s mpox surveillance* demonstrate progress thanks to the DHIS2 Tracker, which has improved case collection and monitoring (50). However, reporting variability, territorial differences, and structural issues affect data quality and usability. The epidemiology is complex, with uneven spatial distribution, sociodemographic disparities, and seasonal patterns. Most reports originate from urban centers like Dakar and Thies, because of their higher population density and better access to healthcare. In contrast, rural areas are underrepresented because of underreporting and logistical challenges. Young adults aged 20–40 are most affected, with a slight male predominance. Incidence peaks during periods of increased mobility and reporting delays. Significant challenges include reporting delays, underreporting in rural areas, and district disparities, often linked to connectivity problems, inadequate training, and supervision (51). These patterns reflect broader African issues, including fragmented health systems, poor interoperability (52), and reliance on manual data, which hinder the transition to integrated digital surveillance. Health alerts are primarily communicated through community relays, which can delay detection of diseases like mpox, especially in underserved regions. This reliance reduces data sensitivity (53) and engagement, which are affected by low health literacy, hierarchical cultures, and a lack of direct reporting options. Community involvement remains limited, leading to delays in critical responses. Shifting to participatory, digital methods with instant reporting tools is crucial for increasing awareness, enabling early detection, and facilitating rapid responses (54).

*The potential of artificial intelligence and mathematical modeling for mpox surveillance:* AI and predictive modeling offer opportunities to integrate heterogeneous data streams, anticipate outbreaks, and optimize resource allocation. The literature supports transforming reactive surveillance into proactive, predictive systems through this approach (55, 56). Studies on mpox have explored methods for identifying vulnerable regions, understanding transmission patterns, and developing testing strategies (57). The convergence of AI and modeling can strengthen health systems (58) but remains underexplored in French-speaking African countries despite recent efforts (59). AI could enhance One Health intelligence by linking human cases, animal reservoirs, and environmental signals to identify early warning trends. Algorithms detect abnormal spatiotemporal patterns in case flows (DHIS2 data, 4S reports, EWARS notifications), allowing early cluster detection before biological confirmation. Machine learning effectively analyzes extensive health datasets (60, 61), as demonstrated during the COVID-19 pandemic by predicting incidence, modeling spread, and facilitating early detection (62). It characterizes transmission dynamics and estimates key epidemiological parameters, including R₀, the basic reproduction number, generation time, and the proportion of asymptomatic cases. However, these methods are rarely used in Africa due to data fragmentation (63). In Senegal, a comprehensive system could be developed by integrating DHIS2 human case data, veterinary reports on rodent populations, mobility data from transport hubs, and environmental indicators such as rainfall and land use. AI algorithms could analyze these diverse data sources daily to identify districts at heightened risk of Mpox spillover or outbreak, forecast potential outbreak trajectories (64, 65) over the next 2 to 4 weeks, and recommend targeted vaccination or awareness campaigns. Additionally, the system could alert surveillance teams to unusual patterns for field verification. By leveraging this predictive, intelligence-driven approach, Senegal could shift from reactive responses to proactive outbreak management, anticipating and containing Mpox more efficiently. However, current mathematical models of mpox have led to a mechanistic understanding of its transmission, pathogenesis, and control (66). Banuet-Martinez et al. (67) suggest integrating within-host and population models, improving data on reservoirs and human behavior, and using models for targeted interventions and scenario planning. Our model uses key epidemiological parameters, accounts for population heterogeneity in risk and contact patterns, and simulates interventions such as vaccination, isolation, contact tracing, and behavioral changes. Sensitivity analyses address uncertainties in within-host dynamics and reservoirs, capturing both mechanistic and population drivers.

*Toward integrated and intelligent surveillance of mpox in Senegal:* The mpox surveillance system (2024–2025) faced structural issues, primarily due to institutional fragmentation and a lack of intersectoral coordination, both of which are crucial for monitoring zoonoses using the One Health approach. Research in Nigeria has shown that 65% of Mpox outbreaks occur in border forest zones with poor surveillance (68), highlighting the need for an integrated animal and environmental approach. African Mpox surveillance also overly focuses on humans, neglecting reservoirs and ecology (69). A setting-based surveillance system, focusing on both humans and animals in their environments, would enable more accurate and efficient outbreak or pandemic prevention (70).

Senegal stands at a crucial turning point, shifting from basic One Health surveillance systems to the cutting edge of intelligent, predictive epidemic intelligence. In line with this, the AI4MPOX-SN project proposes a comprehensive solution comprising three technological components: an integrated platform for human, animal, and environmental data to enhance data sharing; AI to detect signals, predict clusters, and support real-time surveillance, thereby reducing notification delays (71) and mathematical modeling for planning (72). Past Rift Valley fever data indicate that meteorological and entomological information can forecast peaks 4 weeks in advance. The AI4MPOX-SN project offers a proactive surveillance system that automates data collection, leverages predictive analytics, and provides dashboards to support informed decision-making. It encourages community engagement and integrates with existing systems within the One Health framework. Success hinges on strong data governance, coordination, standardized protocols, and skills development (73). Data quality and standardization, especially in regions with limited digital infrastructure (74), affect performance due to fragmented systems and inconsistent practices. Interoperability through APIs, identifiers, and standards is vital.

Ethical considerations are crucial when integrating AI into public health, especially when handling sensitive data such as geolocation and clinical information (75). Transparency, explainable AI (76), data security, and stakeholder education ensure the responsible use of AI. Algorithms should provide clear indicators for decision-makers (77). An AI system for Mpox in Senegal should enhance prediction and response while respecting data sovereignty and cultural context, making technology an ethical and sustainable tool for health justice and local independence. Senegal faces threats such as Rift Valley fever, dengue, and Mpox. A unified platform, like AI4DECLIC-SN, which incorporates tools like *Taggàt, Jottali, and Gëstu*, supports proactive surveillance for multiple diseases. Projects like Epidemic Intelligence from Open Sources (EIOS) (78) demonstrate that technological innovation alone is insufficient without robust governance, data sharing, and an ethical approach to AI (79). Auditing algorithms ensures performance and reduces biases (80). A decolonial approach, involving the co-development of tools with communities, helps prevent global health inequities (81). Local ownership and stakeholder engagement are key, as seen in Kenya’s HIV surveillance (82) and Nigeria’s cholera efforts (83). Co-constructing tools with locals ensures ownership (84) and avoids imposing disconnected solutions (85). Stakeholder involvement at each stage fosters engagement, reduces biases (86), and promotes sustainable innovation (87).

Policy and Global Health Implications: Our findings show that integrating artificial intelligence (AI) and predictive analytics into Senegal’s mpox surveillance system can serve as a scalable model for low- and middle-income countries (LMICs), transforming fragmented data into actionable public health intelligence. Effective epidemic surveillance depends not only on analytical tools but also on resilient digital infrastructure, interoperable data systems, and institutional trust. Developing smart surveillance systems, therefore, requires strengthening governance frameworks, promoting cross-sectoral collaboration, and building sustainable data ecosystems alongside analytical capacity. Senegal’s experience illustrates that a coordinated One Health approach, supported by digital innovation, can generate predictive insights even in resource-constrained settings. This framework provides guidance for other African countries adopting AI-driven surveillance, aligns with the Africa CDC Digital Transformation Strategy, and supports improved regional epidemic preparedness.

Limitations: Several limitations should be considered. Data quality and coverage varied across regions and levels of the surveillance system, potentially affecting the accuracy of descriptive and predictive analyses. In addition, restricted access to real-time data, particularly in rural and remote areas, limits timely monitoring of outbreak dynamics. These constraints highlight the need for continued investments in data infrastructure, equitable surveillance coverage, and capacity-building, which are essential for interpreting findings and guiding future surveillance strategies.

## Conclusion

Senegal’s Mpox surveillance shows progress with the DHIS2 Tracker and the 4S network, which are improving data collection, geolocation, and analysis. However, challenges persist, including notification gaps, underreporting in rural areas, delays, and data quality issues. Community surveillance by intermediaries can miss essential signals, underscoring the need for more effective participatory mechanisms.

Integrating AI with mathematical modeling offers an opportunity to address challenges through early detection and prediction. Their combined use enables proactive surveillance, but it depends on the quality of the data. Without improving literacy, rural coverage, and data protocols, these tools risk bias or incompleteness. Implementation requires a participatory, decolonial approach that prioritizes data sovereignty and incorporates local knowledge. By blending innovation, community involvement, and capacity building, Senegal can develop a resilient system to detect diseases like mpox while maintaining sovereignty. This requires vigilance against dependency, disparities, and biases, supported by ongoing innovation, training, and evaluation. This strategy will enhance surveillance, motivate regional health systems, and promote sustainable and equitable health security in West Africa.

## Data Availability

The original contributions presented in the study are included in the article/supplementary material, further inquiries can be directed to the corresponding author.
